# *Bacillus amyloliquefaciens* alleviates the pathological injuries in mice infected with *Schistosoma japonicum* by modulating intestinal microbiome

**DOI:** 10.3389/fcimb.2023.1172298

**Published:** 2023-05-17

**Authors:** Hao Chen, Ruizheng Sun, Jingyan Wang, Siqi Yao, Syeda Sundas Batool, Zheng Yu, Shuaiqin Huang, Jing Huang

**Affiliations:** ^1^ Department of Parasitology, School of Basic Medical Science, Central South University, Changsha, China; ^2^ Human Microbiome and Health Group, Department of Microbiology, School of Basic Medical Science, Central South University, Changsha, China; ^3^ Department of General Surgery, Xiangya Hospital, Central South University, Changsha, China

**Keywords:** *Bacillus amyloliquefaciens*, *Schistosoma japonicum*, pathological damage, intestinal microbiome, dynamic change

## Abstract

*Schistosoma japonicum* causes serious pathological organ damage and alteration of the intestinal microbiome in the mammalian host, threatening the health of millions of people in China. *Bacillus amyloliquefaciens* has been reported to be able to alleviate the damage to the gut and liver and maintain the homeostasis of the intestinal microenvironment. However, it was unclear whether *B. amyloliquefaciens* could alleviate the hepatic and intestinal symptoms caused by *S. japonicum*. In this study, the intragastric administration of *B. amyloliquefaciens* was performed to treat *S. japonicum*-infected mice during the acute phase. Histopathological analysis and 16S rRNA gene sequencing were used to evaluate the pathological damage and changes in the intestinal microbiome. The results of the study showed that *B. amyloliquefaciens* treatment significantly reduced the degree of granuloma and fibrosis in infected mice. Additionally, recovery of diversity in the intestinal microbiome, decrease in the relative abundance of potential pathogenic bacteria such as Escherichia–Shigella, and reshaping of the interactive network between genera in the intestine were also observed after treatment with *B*. *amyloliquefaciens*. Our findings indicated that treatment with *B. amyloliquefaciens* effectively alleviated the pathological injuries of the liver and intestine in mice infected with *S. japonicum* by modulating the intestinal microbiome, implying that this probiotic can function as an effective therapeutic agent against schistosomiasis. We hope our study will provide auxiliary strategies and methods for the early prevention of schistosomiasis japonica.

## 1 Introduction

Schistosomiasis is a zoonotic parasitic disease that seriously endangers human health. It has been an endemic in 78 countries and regions worldwide, threatening a population of 800 million (McManus et al., 2018). In China, schistosomiasis japonica was mainly prevalent in 12 provinces, municipalities, and autonomous regions in the middle and lower Yangtze River and its south regions (McManus et al., 2010). It has been reported in studies that *Schistosoma japonicum* matures into adults in the intrahepatic portal system and then migrates in pairs to mesenteric veins, where it copulates and lays eggs in the blood vessels of the intestinal wall. Therefore, diarrhea, fatigue, and anemia could be caused by schistosomiasis japonica in the early stage of infection and portal hypertension syndrome, ascites, and liver fibrosis in the late stage (Colley et al., 2014). It generally takes 23–35 days from the time of transcutaneous infection with cercariae to produce eggs by mating. Therefore, the exacerbation phase of schistosomiasis japonica would appear in 4–5 weeks, and this stage is called the acute phase of *S. japonicum* infection (Li et al., 2018). Studies revealed that mature eggs contain *S. japonicum* miracidium, which secretes cytolytic material that penetrates through the eggshell into the intestinal mucosa, damaging the vessel wall to form granulomas and breaking the surrounding intestinal mucosal tissue into necrosis (Barnett, 2018). It was found previously that due to intestinal peristalsis and an increase in intra-abdominal pressure with intravascular pressure, worm eggs with necrotic tissue fall into the intestinal lumen and get discharged with the feces. The symptoms of intestinal granuloma caused by *S. japonicum* infection are similar to those of inflammatory bowel disease (IBD), typically characterized by hematochezia and unformed stools (McManus et al., 2018).

It was observed that the bacterial community of the gut constitutes the largest microbiota in the human body, and its homeostasis and balance are essential for human health. Disturbance of the intestinal microbiota can easily cause diarrhea, IBD, and even increase the risk of colorectal cancer (CRC) (Choi et al., 2017). Previous studies have shown that alterations in the intestinal microenvironment caused by granulomas could disrupt the stability and composition of the bowel microbiome (Zhao Y. et al., 2019). *Odoribacter* and *Lachnospiraceae UCG-001* have been observed to become more abundant after infection, and *Alistipes* might be potential biomarkers that could produce anti-inflammatory metabolites to promote the differentiation of anti-inflammatory Treg/Tr1 cells in the gut of mice (Dziarski et al., 2016; Li et al., 2016; Hu et al., 2020). Furthermore, studies reported that the composition of the intestinal microbiota was distinct during the acute and chronic phases of *S. japonicum* infection (Zhou et al., 2022). Previous researchers found that gut microbiota changes are capable of ameliorating the pathological conditions caused by *S. japonicum*. By depletion of the gut microbiome in infected mice by antibiotics and its cohousing with healthy mice, researchers discovered that microbiome changes in the gut of infected mice indeed attenuated intestinal granuloma formation as well as the fibrotic response and reduced inflammatory factor levels (Zhang et al., 2020). These findings illustrated that modification of the intestinal microbiome can effectively alleviate the symptoms of *S. japonicum* infection, indicating a close relationship between *S. japonicum* infection and the host intestinal microbiome.

It has been stated in previous studies that praziquantel has been used clinically as the first-choice drug for the prevention and treatment of schistosomiasis japonica (Yahya et al., 2023), but despite being a broad-spectrum anti-helminth drug, it faced several challenges, including high side effects, low solubility, low efficacy against *S. japonicum* juveniles, and resistance development in *S. japonicum* caused by long-term and massive use (Li et al., 2011). Therefore, the development of new anti-schistosomal drugs and the implementation of more effective means of prevention and treatment appear to be extremely important and urgent. Probiotics could maintain the balance of the intestinal microbiota and possess anti-inflammatory and anti-cancerous activity, defined by the World Health Organization as living microorganisms that benefit the health of the host when administered in adequate amounts (Guarino et al., 2015). At present, probiotics are employed clinically as a therapeutic approach to prevent intestinal diseases. Through early prevention and treatment with probiotics, the symptoms of intestinal diseases and the disorders of the microbiota can be alleviated, and the homeostasis of the gut microbiome can be inextricably improved and optimized to maintain a normal state of function (Sanders et al., 2011). Intervention with probiotics in the treatment of schistosomiasis japonica is a therapeutic advancement. It has been proven by earlier studies that gavage of probiotics effectively alleviates the symptoms of *S. japonicum* infection, as *Bacillus subtilis* reduces pathological damage and regulates gene expression in the host mice (Lin et al., 2021).

*Bacillus amyloliquefaciens*, a bacterium with probiotic properties, has been introduced to livestock feed on an industrial level. It was added to reduce poultry mortality by reducing the impact of pathogenic bacteria on their gut (Abriouel et al., 2011). Recently, it was found that the number of *B. amyloliquefaciens* in dog intestines was significantly increased, whereas *Escherichia coli* decreased significantly after gavage with *B. amyloliquefaciens*, indicating that *B. amyloliquefaciens* has intestinal adhesive and antimicrobial activity (Vazquez-Mendoza et al., 2018). Moreover, *B. amyloliquefaciens* was found to ameliorate pathological damage in mice fed a high fiber diet by reducing the degree of obesity and inflammation as well as alleviating liver injury (Wang et al., 2019). In addition, *B. amyloliquefaciens* treatment was reported to promote the clearance of *E. coli* in RAW 264.7 cells by activating autophagy and to produce anti-inflammatory effects by reducing the level of JNK phosphorylation caused by *E. coli* (Wu et al., 2017). In general, *B. amyloliquefaciens* can modulate the homeostasis of the intestinal microbiota, reduce the level of intestinal inflammation, and reduce the degree of liver injury. However, it was unclear whether *B. amyloliquefaciens* could alleviate the symptoms caused by *S. japonicum* infection.

In this study, we performed intragastric administration of *B. amyloliquefaciens* to treat mice acutely infected with *S. japonicum* and then evaluated its effects on hepatic and intestinal injuries. Additionally, 16S rRNA gene sequencing was conducted to analyze the changes in gut microbiota in infected mice post treatment with *B. amyloliquefaciens*. Our work demonstrated that treatment with *B. amyloliquefaciens* during the acute phase could alleviate the pathological injuries in mice infected with *S. japonicum*. We aimed to provide an effective agent for the early prevention and treatment of *S. japonicum* infections.

## 2 Methods

### 2.1 Preparation for suspensions of *B. amyloliquefaciens*

*B. amyloliquefaciens* was isolated from healthy human feces and identified by using full-length primers of 16S rRNA through NCBI-Blast. Approximately 0.5 g of human feces were diluted in a 40 ml PBS solution, followed by water bathing at 80°C for 20 min, and then left to cool down at room temperature. Next, 100 μl of the supernatant was aspirated into lysogeny broth (LB) plates and spread, followed by incubation at 37°C. Subsequently, single colonies were picked according to the bacterial morphology and streaked for passaging. Single colonies were isolated after three passages of culture. The extraction of single-colony DNA was performed as per the protocol of the universal genomic DNA kit (Cwbio, Jiangsu, China). Afterward, the full-length 16S rRNA gene was amplified by polymerase chain reaction (PCR) using the forward primers 27F (5’-AGAGTTTGATCMTGGCTCAG-3’) and reverse primers 1492R (5’-GGTTACCTTGTTACGACTT-3’) and sequenced by first-generation sequencing. Finally, *B. amyloliquefaciens* was identified by NCBI-Blast. Moreover, *B. amyloliquefaciens* was stored at −80 °C for further culturing.

One day before intragastric administration, *B. amyloliquefaciens* was activated and cultured in LB medium at 37 °C and 200 rpm in the constant-temperature shaker until its OD_600_ reached 0.8–1.0. Intragastric administration was performed on each mouse with a 0.3-ml suspension of *B. amyloliquefaciens* every 3 days at a fixed time. The bacterial suspension was refreshed every time before intragastric administration.

### 2.2 Mice infection and intragastric administration of *B. amyloliquefaciens*

**Figure 1:**
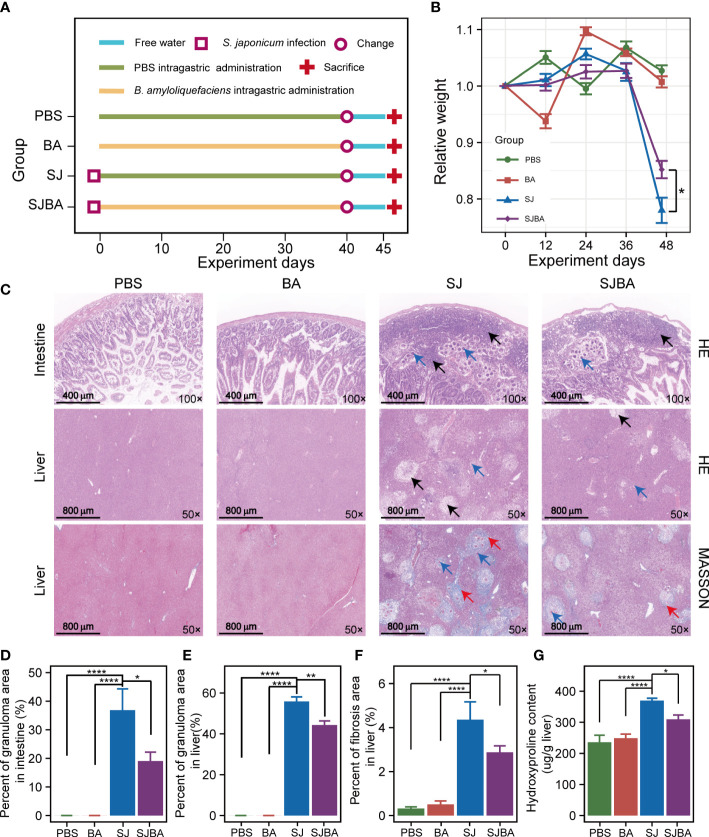
Treatment of *B*. *amyloliquefaciens* alleviated *S. japonicu-*induced hepatic and intestinal granulomas in mice. **(A)** Experimental design schedule for the study. **(B)** Changes in relative body weight among the four groups. Seven mice were included from each group for calculation on days 0, 12, 24, and 36. Meanwhile, seven mice in the PBS group, seven mice in the BA group, four mice in the SJ group, and five mice in the SJBA group were included in the calculation on the 45th day. Data were analyzed by one-way analysis of variance (ANOVA) and Fisher’s least significant difference (LSD) multiple comparison posttest. **(C)** H&E staining and Masson’s trichrome staining of the small intestine and liver. The numbers in the lower right represent the magnification of images. The black arrows indicated granulomas. The red arrows indicated fibrosis. Blue arrows indicated eggs of *S. japonicum*. **(D)** The percentage of granuloma area in the small intestine. **(E)** The percentage of granuloma area in the liver. The data were analyzed by the Kruskal–Wallis test. **(F)** The percentage of fibrosis area in the liver. The data were analyzed by one-way ANOVA and LSD’s multiple comparison posttest. **(G)** The hydroxyproline content in the liver. Data were analyzed by one-way ANOVA and LSD’s multiple comparison posttest. *P <0.05; **P <0.01; ****P <0.0001.

A total of 28 female pathogen-free BALB/c mice (approximately 5 weeks old) were purchased from Hunan Sleek Jingda (SLAC), Changsha, China. All mice were settled in plastic cages with metal fence covers. The environmental conditions were sterile, with free access to standard rat food and drinking water under controlled temperature (25 ± 5°C), humidity (60%–70%), and light (12/12-hour light/dark cycle). After adaptive feeding, mice (approximately 7 weeks old, body weight: 20 ± 2 g) were separated into four groups randomly: PBS, healthy mice with intragastric administration of PBS (phosphate buffered saline); BA, healthy mice with intragastric administration of *B. amyloliquefaciens*; SJ, *S. japonicum*-infected mice with intragastric administration of PBS; and SJBA, *S. japonicum* infected mice with intragastric administration of *B. amyloliquefaciens*. Each group contained seven mice. Based on the design of the experiment (**Figure 1A**), mice in the SJ and SJBA groups were infected with 27 ± 3 *S. japonicum* cercariae *via* shaved abdominal skin. The PBS and BA groups served as controls without any treatment for infection. All the mice were returned to original environment to house after infection. Intragastric administration of PBS and *B. amyloliquefaciens* started one week before infection and was conducted according to the design of the experiment (**Figure 1A**) after infection.

### 2.3 Sample collection

The body weight of all mice was measured once every 12 days. Fecal samples of all mice were collected once every 12 days and stored at −80 °C for further analysis. On day 45 after infection, all mice were sacrificed by chloral hydrate asphyxiation and cervical dislocation. The collection of fecal samples and the measurement of weight were conducted one day before sacrifice. Whole liver and spleen samples were collected to measure weight. The left liver lobes and small intestines were fixed in 4% paraformaldehyde for histopathological analysis. The remaining liver samples were assayed for hydroxyproline content by following the protocol of the hydroxyproline content assay kit (Solarbio, Beijing, China).

### 2.4 Histological staining

The samples of livers and intestines were used for histopathological analysis. Each group contained three livers and three small intestines for analysis. The slices of livers and intestines were subjected to H&E (hematoxylin–eosin) staining and Masson’s trichrome staining. Images of slices were captured and scanned by a Pannoramic panoramic slide scanner (3DHISTECH, Hungary). The whole tissue area and the blue-positive region were analyzed using Image-Pro Plus 6.0 software (Media Cybernetics, USA) and CaseViewer 2.4 software (3DHISTECH, Hungary). The percentage of the fibrotic area was calculated based on the area of the blue-labeled region/the total area of the whole liver tissue. Granulomatous responses in the intestine and liver were estimated by the ratio of granulomas to the area of the whole tissue.

### 2.5 DNA extraction and high-throughput sequencing of 16S rRNA

Three samples from each group at different sampling time points were selected for 16S rRNA sequencing. Total genomic DNA from fecal samples was extracted by following the protocol of the OMEGA Soil DNA Kit (M5636-02) (Omega Bio-Tek, Norcross, GA, USA) and stored at −20°C prior to further analysis. The quantity and quality of extracted DNA were measured using the NanoDrop NC2000 spectrophotometer (Thermo Fisher Scientfic, Waltham, MA, USA) and agarose gel electrophoresis, respectively. The V3–V4 region of the 16S rRNA gene was subsequently amplified by polymerase chain reaction (PCR) using the forward primers 338F (5’-ACTCCTACGGGAGGCAGCA-3’) and reverse primers 806R (5’-GGACTACHVGGGTWTCTAAT-3’). The qualified libraries were then sequenced on the Illumina NovaSeq platform with the NovaSeq-PE250 sequencing strategy at Shanghai Personal Biotechnology Co., Ltd. (Shanghai, China). After sequencing, the data was decomposed into appropriate samples based on barcodes, and the appropriate sequences were imported into downstream software. Quality control and denoising of raw reads were performed based on the standard amplicon pipeline as described previously (Liu et al., 2021). The feature table and taxonomy annotation table were used for further data analysis.

### 2.6 Data analysis

All statistical analysis was performed using R (version 4.2.1) (R Core Team, 2022). The statistical results were visualized by the “ggplot2” package unless specified otherwise (Wickham, 2016). Shannon index and beta diversity were calculated by the “vegan” package (Oksanen J et al., 2022). The survival curves of mice were performed by packages of ‘survival’ and ‘survminer’ (Therneau and Grambsch, 2000; Alboukadel Kassambara and Przemyslaw, 2021). The analysis of constrained principal coordinate analysis (CPCoA) based on Bray–Curtis distance was performed by the “amplicon” package (Liu Y and Chen, 2021). In addition, genera with significant changes in relative abundance were discovered by Linear Discriminant Analysis (LDA) Effect Size (LEfSe) (http://huttenhower.sph.harvard.edu/galaxy/) (Galaxy, 2022). The threshold of significance was set at 0.05, while the threshold of the LDA score was set at 3.5. The analysis of Spearman correlation for genera with significant changes in relative abundance was calculated by the “Hmisc” package (F, 2022) and visualized by the “pheatmap” package (Kolde, 2019). The network showed that interactions between genera were established based on Spearman correlation analysis by the “igraph” package (Gabor Csardi, 2016) and Gephi software (version 0.10.0) (Bastian M. and Jacomy, 2009). The threshold of the *P*-value corrected by the Benjamin and Hochberg false discovery rate (FDR) was 0.05.

## 3 Results

### 3.1 *B. amyloliquefaciens* treatment significantly alleviates the pathological injuries in mice infected with *S. japonicum*

To determine the effects of *B. amyloliquefaciens* on *S. japonicum*-infected mice, the experiment was designed on schedule for parasitic infection (**Figure 1A**). It was reported that loss of body weight in mice is a common feature of *S. japonicum* infection. Therefore, the changes in relative weight of the mice were recorded and analyzed during the experiment. The results demonstrated that there was no significant difference in the relative weight of mice among the four groups within 36 days post-infection, but it decreased dramatically after 36 days in the SJ and SJBA groups compared to the PBS and BA groups, indicating that infected mice began to show obvious pathological responses after 36 days in this experiment (**Figure 1B**). Although the weight loss of mice caused by *S. japonicum* infection is dramatic, even below 20%, we found that treatment with *B. amyloliquefaciens* significantly prevented weight reduction. At the same time, we found that the intervention of *B. amyloliquefaciens* also reduced the mortality of infected mice (**Supplementary Figure S1**). Visceral enlargement and fibrosis are typical pathological injuries in schistosomiasis japonica. Here, we found that the liver and spleen of mice in the PBS and BA groups appeared pink and soft in texture but became enlarged, black, and brittle at 45 days post-infection with *S. japonicum* (**Supplementary Figure S2A**). A sharp increase in the weight of the liver and spleen has been observed in the SJ group, which significantly decreased after *B. amyloliquefaciens* intervention (**Supplementary Figures S2B, C**).

In addition, histopathological analysis was performed to assess the degree of fibrosis and granulomas. According to the results of H&E staining, we found that eggs of *S. japonicum* and granulomas were found in the submucosa of the SJ and SJBA groups, affecting the integrity of the mucosa compared to the small intestine of the PBS and BA groups (**Figure 1C**). But the percentage of granulomatous area significantly declined both in the liver and small intestine in the SJBA group after treatment with *B. amyloliquefaciens* (**Figures 1D, E**). In the results of Masson’s trichrome staining, the percentage of fibrosis area in the liver decreased significantly in the SJBA group (**Figure 1F**). Subsequently, we found the content of hydroxyproline, which is an index of liver fibrosis, decreased significantly in *S. japonicum*-infected mice after treatment with *B. amyloliquefaciens* (**Figure 1G**). These results indicated that the use of *B. amyloliquefaciens* as a treatment can reduce *S. japonicum*-induced hepatic and intestinal granulomas and fibrosis, thereby protecting mice from infection.

### 3.2 *B. amyloliquefaciens* treatment modulates the dynamic changes of diversity and bacterial composition of gut microbiota

**Figure 2:**
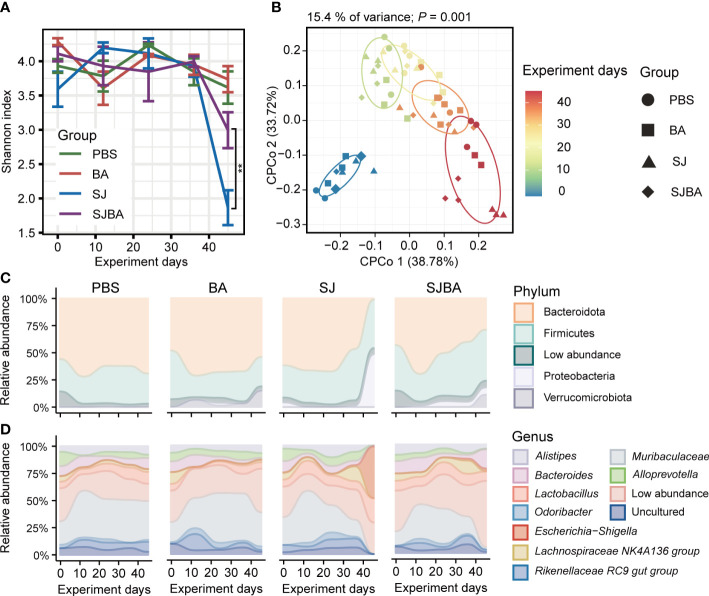
Treatment of *B*. *amyloliquefaciens* altered the diversity and composition of the gut microbiota of *S. japonicum-*infected mice. **(A)** The changes in the Shannon index with the progress of the experiment in four groups. **(B)** Analysis of CPCoA among the four groups. The color bar represented experimental days. Different shapes distinguished the four groups. **(C)** The changes in relative abundance of phyla in four groups during the experiment. **(D)** The changes in relative abundance of genera in four groups during the experiment. The X-axis represented the experimental days. The Y-axis represents the relative abundance of different phyla or genera. Modules in different colors separated different phyla or genus. “Low abundance” represents a genus in low relative abundance that cannot be clearly shown in the figure.

To evaluate the homeostasis and composition of the bacterial community, we analyzed the dynamic changes in diversity and bacterial composition in the gut of mice among four groups. Alpha diversity was presented by the Shannon index, and we found that the trend of the Shannon index was the same as the changes in relative body weight. There was no significant difference before 36 days post-infection within the four groups, but it declined dramatically on day 45 in the SJ group (**Figure 2A**). Furthermore, the results obtained from differential analysis by Shannon index on day 45 showed that infection with *S. japonicum* caused a sharp decline in Shannon index, which can be reversed by treatment with *B. amyloliquefaciens* (**Supplementary Figure S3A**). To clarify the variations among samples following the experimental time, we performed CPCoA analysis based on the Bray–Curtis distance. We found that the distance between each two samples at every time point became larger, and the distance between each time point was also significantly distinguished (**Figure 2B**). The results from CPCoA analysis on day 45 showed that PBS and BA groups were closer in distance, and *S. japonicum* infection led to a larger distance between PBS and BA groups, while administration of *B. amyloliquefaciens* resulted in a larger distance compared to the SJ group. (**Supplementary Figure S3B**). The Bray–Curtis distance between each time point and day 0 was calculated, and a linear fit was performed. Our study observed that the distance increased dramatically with the passage of time in the SJ group, while the SJBA group showed stable trends at the end of the experimental day (**Supplementary Figure S3C**). These results indicated that the intervention of *B. amyloliquefaciens* reshapes the composition of the bacterial community and helps reduce variations among samples after *S. japonicum* infection.

To elucidate how the treatment with *B. amyloliquefaciens* influenced the composition of the bacterial community in *S. japonicum*-infected mice, we analyzed the composition of the microbiome at the levels of phylum and genus. Significant changes were observed in bacterial composition after 36 days of infection but not before this period. Contrary to the PBS and BA groups, on day 45, the relative abundance of the phylum Bacteroidota dropped vividly, while the dominance of Proteobacteria displayed a significant rise in the SJ group, but this tends to return to normal levels in the SJBA group (**Figure 2C**). By compositional analysis at the level of genera, we observed an obvious increase of up to 50% in the relative abundance of Escherichia–Shigella in the SJ group and a clear decrease in Muribaculaceae to almost 0% on day 45 in the SJ group. Meanwhile, in the SJBA group, the relative abundance of Escherichia–Shigella dropped to the same level as the PBS and BA groups, but the abundance of Bacteroides increased (**Figure 2D**).

### 3.3 The relative abundance of *Muribaculaceae* and *Escherichia–Shigella* changed most significantly during exacerbations of acute phase

**Figure 3:**
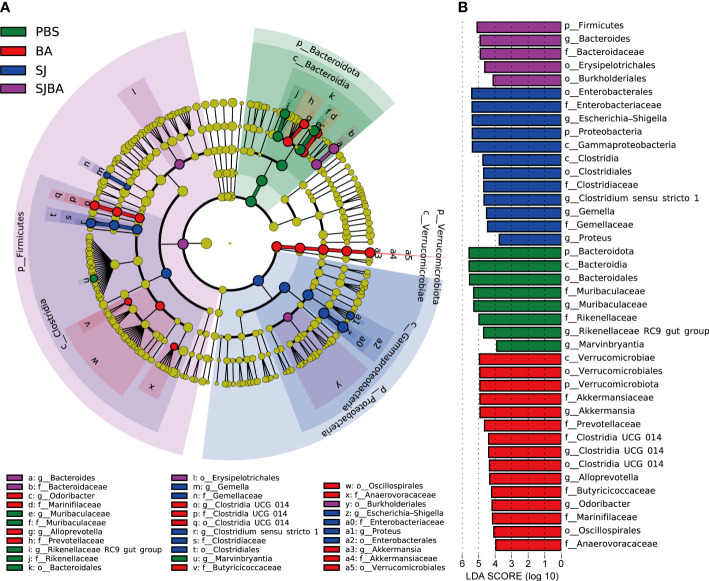
Genera with significant changes in the relative abundance in the gut of *S. japonicum*-infected mice after treatment with *B*. *amyloliquefaciens*. **(A)** A cladogram showing the discriminated taxa in different groups. Regions with different colors represented different groups. Nodes in different colors represented significant changes in relative abundance in different groups. The tellow nodes indicated no significant changes in the corresponding group. **(B)** A histogram with linear discriminant analysis (LDA) scores in four groups. Taxa highlighted in different colors indicate overrepresentation in the corresponding groups. The threshold of significance is set at 0.05. The threshold of the LDA score is set at 3.5.

As indicated by the above results, day 36 proved to be a key time point for the progression of schistosomiasis japonica. Nearly all the pathological responses and changes in the homeostasis of the bacterial community worsened between days 36 and 45 post-infection. Thus, LEfSe analysis was performed to explain which bacteria had significant changes in their relative abundance from levels of phylum to genus between days 36 and 45. The threshold of adjusted *P* and LDA score was 0.05 and 3.5. The findings demonstrated that the phyla Bacteroidota and Proteobacteria were the most significantly altered ones. (**Figure 3B**). At the genus level, *Escherichia–Shigella*, *Clostridium sensu stricto 1*, *Proteus*, and *Gemella* had significant increases in their relative abundance in the SJ group. Bacteroides was significant in the SJBA group with increased relative abundance (**Supplementary Figure S4**). Meanwhile, *Muribaculaceae*, *Marvinbryantia*, and *Rikenellaceae RC9 gut groups* were significantly associated with the PBS group, and *Akkermansia*, *Alloprevotella*, *Odoribacter*, and *Clostridia UCG 014* were significantly associated with the BA group (**Figure 3B**). The relative abundance of all the genera related to the PBS and BA groups dropped almost to 0% in the SJ group but slightly resurged in the SJBA group (**Supplementary Figure S4**). In terms of relative abundance, *Muribaculaceae* and *Escherichia–Shigella* were the most altered genera. The cladogram showed the most relevant clades among groups, which was in accordance with the above-mentioned results (**Figure 3A**).

### 3.4 Intervention of *B. amyloliquefaciens* reshaped the interaction network between genera with significant changes in relative abundance

**Figure 4:**
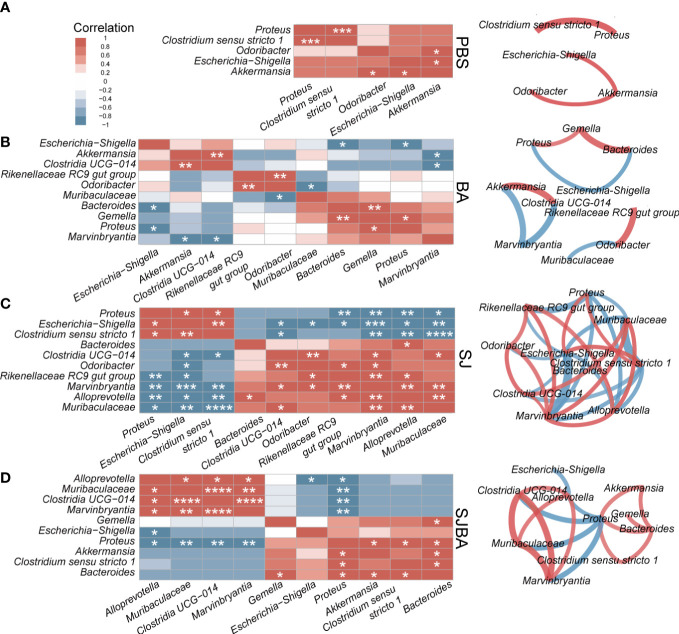
The analysis of correlation and network among the genera with significant differences in relative abundance. Correlation and network analysis among the genera with significant differences in relative abundance was performed among the groups of PBS **(A)**, BA **(B)**, SJ **(C)**, and SJBA **(D)**. Different colors represented positive or negative correlations. The intensity of the color represented the strength of the correlation. Edges in different colors between two genera represent the positive or negative correlation. The thickness of the edges represents the strength of the correlation. *P <0.05; **P <0.01; ***P <0.001; ****P <0.0001.

To illustrate the relationship and interaction of genera in terms of changes in relative abundance among groups, we performed Spearman correlation and network analysis. Genera included in the correlation analysis were selected by the LEFSe analysis (**Figure 3**). All genera had significant differences in relative abundance under the screening based on the set threshold (refer to *Methods*). Spearman correlation was used to calculate the correlation between the picked genera. Then, genera with significant correlations were picked out to reconstruct the correlation heatmap figure for easy interpretation by readers. In the meantime, those selected genera were also used to perform an analysis of the interaction network. As suggested by the results of Spearman correlation, the significant correlation between genera in the SJ and SJBA groups was notably more complex than in the PBS and BA groups (**Supplementary Figure S5**). In the PBS and BA groups, genera with significant correlations were relatively few, and the correlation network was simple (**Figures 4A, B**). In the SJ group, there was a significant positive correlation between *Proteus*, *Escherichia–Shigella*, and *Clostridium sensu stricto 1*. The same correlation was also shown between *Clostridia UCG 014*, *Odoribacter*, *Rikenellaceae RC9 gut group*, *Muribaculaceae*, *Marvinbryantia*, and *Alloprevotella* in the SJ group. It was noted that *Muribaculaceae*, *Marvinbryantia*, and *Alloprevotella* had a highly significant negative correlation with *Proteus*, *Escherichia–Shigella*, and *Clostridium sensu stricto 1*. Especially, *Escherichia–Shigella* exhibited significant negative correlations with other genera that were significantly associated with the PBS and BA groups (**Figure 4C**). After treatment with *B. amyloliquefaciens*, the original correlation network was altered. *Muribaculaceae*, *Marvinbryantia*, and *Clostridia UCG 014* established an extremely positive correlation with each other. Proteus showed a negative correlation with *Bacteroides* and *Clostridium sensu stricto 1* while presenting a positive correlation with *Alloprevotella*, *Muribaculaceae*, *Marvinbryantia*, and *Clostridia UCG 014* (**Figure 4D**).

## 4 Discussion

*S. japonicum*, the only human blood fluke present in China, severely threatens the health of millions of people and affects socioeconomic development (Xue et al., 2022). It was stated earlier that organ edema, fibrosis of the liver, and granulomas in intestinal tissues could be caused by *S. japonicum* infection (Yang et al., 2021). Furthermore, the homeostasis and composition of the intestinal bacterial community were reported to be disrupted by alterations in the gut microenvironment caused by intestinal granulomas (Zhou et al., 2020). Studies suggested there was no target drug for treating schistosomiasis japonica except praziquantel, which is a broad-spectrum anti-helminthic drug (Wang et al., 2022). Previous studies suggested that changes in the gut microbiome reduce the symptoms caused by *S. japonicum* infection (Zhang et al., 2020). Therefore, we performed this study to evaluate whether intragastric administration of *B. amyloliquefaciens* could also alleviate the pathological injuries in mice infected with *S. japonicum* during the acute phase or not. Our results demonstrated that the employment of *B. amyloliquefaciens* was able to relieve *S. japonicum*-induced hepatic as well as intestinal granulomas and reshape the bacterial community in the intestine of *S. japonicum-*infected mice. Additionally, the diversity and composition of the intestinal microbiome were gradually returned to normal levels after treatment with *B. amyloliquefaciens*. Moreover, the relative abundance of *Escherichia–Shigella* was significantly reduced in the SJBA group, which was observed to be greatly elevated in the acute phase of *S. japonicum* infection. Furthermore, the interaction network among genera was also reshaped in the SJBA group, contrary to that in the SJ group.

Fibrosis of the liver and intestinal granulomas were the typical symptoms caused by the *S. japonicum* infection (Gunda et al., 2020). An earlier study stated that *S. japonicum* matures into an adult worm in the hepatic portal vein and subsequently migrates in pairs to the mesenteric veins to clasp and lay eggs. Eggs were regarded as one of the main pathogenic factors in schistosomiasis japonica (Burke et al., 2009). It was found that eggs of *S. japonicum* induce a granulomatous immune response in the host, which is largely characterized by lymphocytes, eosinophils, and alternatively activated macrophages (Pearce and MacDonald, 2002; Fairfax et al., 2012). By observing the pathological response in the acute phase of *S. japonicum* infection, weight loss can be significantly reversed after treatment with *B. amyloliquefaciens*. Furthermore, the degree of liver fibrosis as well as the granuloma area of the liver and small intestine were also observed to be significantly reduced after treatment with *B. amyloliquefaciens*. These results indicate that treatment with *B. amyloliquefaciens* can relieve the hepatic and intestinal fibrosis and granuloma in the acute phase of schistosomiasis japonica. *B. amyloliquefaciens* was widely used in food processing as a probiotic. Some of its primary and secondary metabolites, like lipopeptides, have anti-inflammatory and anticancerous effects, thus protecting the mucosal layer of the gut (WoldemariamYohannes et al., 2020). According to other studies, *B. amyloliquefaciens* SC06 could reduce the levels of JNK phosphorylation triggered by *E. coil*, thereby preventing acute inflammation (Wu et al., 2017; Wang et al., 2019). Therefore, there might be relationships between metabolites of *B. amyloliquefaciens* and host immune responses caused by granuloma, but further research is needed. Consequently, our results demonstrated that treatment with *B. amyloliquefaciens* can effectively attenuate the pathological damage caused by *S. japonicum* infection, indicating that it can act as a potential therapeutic agent for schistosomiasis, but further investigation is required.

The intestinal microbiome was found to have a close association with infection by *S. japonicum*. *S. japonicum* infection was found to cause dysbiosis of the gut microbiome, while modifications in the microbiota would alleviate the symptoms caused by *S. japonicum* infection (Zhang et al., 2020). Alpha diversity presented the number, category, and abundance of microbiomes within groups, and beta diversity such as Bray–Curtis distance refers to microbiota differences between samples or groups, which helped to understand whether differences in microbiota composition between two groups were significant (Qian et al., 2020). Our results showed that dysbiosis of gut microbiota caused by *S. japonicum*, manifested itself as rapid decline in alpha diversity and sharp increase in difference of bacterial composition in samples within groups, along with a steady rise and domination in the relative abundance of some phyla and genera, which were in accordance with the results of previous studies (Hu et al., 2020). By treating with *B. amyloliquefaciens*, the variation in alpha diversity, beta diversity, and bacterial composition were significantly improved. The results indicated that employment of *B. amyloliquefaciens* reshaped the intestinal ecology, improved the homeostasis of microbiome, and avoided the simplification of bacterial composition. Previous studies have shown that the intervention of *B. amyloliquefaciens* can reduce the levels of inflammatory factors and resume the dysbiosis of microbiota in the DSS-induced enteritis model (Cao et al., 2018). In addition, early colonization by *B. amyloliquefaciens* alleviated jejunal villus damage with reduced expression of proapoptotic genes in a study of enteritis caused by *Clostridium perfringens* (Jiang et al., 2021). Thus, combined with the results of histological staining, we found that there might be a potential correlation between amelioration of dysbiosis in microbiota and attenuation of pathological responses. The changes of intestinal microbiota due to treatment with *B. amyloliquefaciens* might be one of the reasons why schistosomiasis japonica was alleviated.

According to the results, the phenotype of mice from days 36 to 45 was completely different from that during the first 36 days, particularly manifested by a sudden drop in diversity of the microbiota and a trend toward simplification of bacterial composition. The analysis of LEfSe using samples between days 36 and 45 was performed to evaluate dysbiosis in the microbiota caused by *S. japonicum* infection. The characteristic of disturbance in the gut microbiota refers to changes in the composition of the microbiota associated with the host’s disease state (Ni et al., 2017). Our results showed that the infection of *S. japonicum* led to a significant increase in the relative abundance of *Escherichia–Shigella*, *Clostridium sensu stricto 1*, *Proteus*, and *Gemella*. Exclusively *Escherichia–Shigella*, rapidly increased to nearly 50% in relative abundance. Meanwhile, we observed a sharp decline in relative abundance of *Muribaculaceae*, *Marvinbryantia*, *Rikenellaceae RC9 gut group*, *Akkermansia*, *Alloprevotella*, *Odoribacter*, and *Clostridia UCG 014*, which fell to nearly 0% in the SJ group. Interestingly, the relative abundance of *Escherichia–Shigella* and *Muribaculaceae* was the most variable. *Escherichia–Shigella* has a strong association with IBD. As a potential biomarker of dysbiosis of the gut microbiome, it could stimulate the secretion of IL-6 and TNF-α, promoting inflammatory responses (Berry et al., 2015; Di Lorenzo et al., 2019). Therefore, a rise in the relative abundance of *Escherichia–Shigella* has a positive correlation with the disease severity in ulcerative colitis (UC) patients (Kojima et al., 2012). Muribaculaceae, a potentially beneficial bacterium, could modulate immune cells and decrease proinflammatory factor levels, and it has been proven to be a promising agent in alleviating and treating IBD (Qi et al., 2017; Mo et al., 2022; Niu et al., 2022). However, *Clostridium sensu stricto 1*, *Proteus*, and *Gemella* have been proven to have a role in accelerating the occurrence of IBD (Lopez-Dupla et al., 1996; FitzGerald et al., 2006; Garrett et al., 2010; Wen et al., 2021). Inclusively, *Marvinbryantia*, *Rikenellaceae RC9 gut group*, *Akkermansia*, *Alloprevotella*, *Odoribacter*, and *Clostridia UCG 014* were all potential beneficial bacteria that could promote the synthesis of short-chain fatty acids (SCFAs) with the function of inhibiting the expression of inflammatory factors (Gao et al., 2022; Hu, 2022; Leibovitzh et al., 2022; Cai et al., 2023; Gao et al., 2023; Han et al., 2023). Therefore, the dramatic changes in *Escherichia-Shigella* and *Muribaculaceae* relative abundance might be one of the main reasons for deterioration and death from days 36 to 45. Moreover, a quantitative increase in *Clostridium sensu stricto 1*, *Proteus*, and *Gemella* and a decrease in potential beneficial bacteria boosted this trend. After treatment with *B. amyloliquefaciens*, the relative abundance of *Escherichia–Shigella* sharply dropped, accompanied by decrease in the relative abundance of other potentially harmful bacteria. Many studies have shown that *B. amyloliquefaciens* was able to produce bacteriocin-like inhibitory substances (BLIS), which had antimicrobial activity against pathogenic bacteria like *Clostridium perfringens*, *E. coli* and *Yersinia* (Abriouel et al., 2011). Therefore, BLIS might be an important factor that could cause a decrease in the relative abundance of pathogenic bacteria, thereby attenuating the pathological response in *S. japonicum*-infected mice. We found a notable increase in the relative abundance of Bacteroides in the SJBA group, which was known to significantly increase colonic inflammation in UC and modulate inflammatory responses by modulating Treg cell responses (Bloom et al., 2011; Zhao H. et al., 2019). Consequently, the elevated relative abundance of Bacteroides and the failure to reverse the relative abundance to a normal level in the case of beneficial bacteria might explain the reason for the death of infected mice even after treatment with *B. amyloliquefaciens*. The results from the analysis of LEfSe demonstrated that the relative abundance of genera significantly changed during the exacerbation phase. Meanwhile, the intervention of *B. amyloliquefaciens* reshapes the composition of the bacterial community and might reduce the relative abundance of potential pathogenic bacteria by secreting BLIS. These results indicated that *B. amyloliquefaciens* treatment could alleviate the pathological responses induced by *S. japonicum* infection by ameliorating the disturbance of the gut microbiota.

To elucidate the relationship among the genera with significant changes in relative abundance, we performed an analysis of correlation. In the PBS and BA groups, the interaction network among genera was relatively simple. Potential beneficial bacteria dominated, while potential pathogenic bacteria had a relatively low relative abundance, resulting in the stability of the intestinal microenvironment. In the SJ group, the interaction network became more complex. The relative abundance of *Escherichia–Shigella*, *Clostridium sensu stricto 1*, and *Proteus* increased with significant positive relationships among each other. Our results showed the sum of the relative abundances of the three potential harmful bacteria was close to 60%. *Escherichia–Shigella* had a significant negative correlation with almost all potential beneficial bacteria. Therefore, it was difficult to effectively inhibit pathogenic bacteria due to the low relative abundance of beneficial bacteria and the dominant position of *Escherichia–Shigella*. The intervention of *B. amyloliquefaciens* changed the interaction network relationships among the genera in the gut microbiota. Specifically, the positive correlation among potential pathogenic bacteria was destroyed with the decrease in the relative abundance of *Escherichia–Shigella*. Moreover, the relative abundance of all potential beneficial bacteria increased in positive correlation with each other. The results suggested that the symbiotic relationship among beneficial bacteria may be one of the key factors in alleviating the severity of disease in infected mice. It is worth noting that the relative abundance of Bacteroides also increased and maintained a significant positive correlation with other potential pathogenic bacteria except *Escherichia–Shigella*, which may be the reason of mice deaths even after receiving *B. amyloliquefaciens* as a treatment. Through our analysis, we found that the symbiotic and competitive relationship among bacteria with significant changes may affect the severity and outcome of symptoms in *S. japonicum*-infected mice. It has also been indicated that the intervention of *B. amyloliquefaciens* could destroy the mutually promoting relationship among potential pathogenic bacteria and promote the positive relationship among potential beneficial bacteria; eventually, it might improve the pathological reaction of *S. japonicum*-infected mice.

In general, treating mice with *B. amyloliquefaciens* could alleviate the pathological reaction in mice infected with *S. japonicum*. In addition, it also reshapes the intestinal microbiome to achieve a stable state by resuming the diversity of gut flora, reducing the relative abundance of *Escherichia–Shigella*, and intervening in the correlation between potential pathogenic genera and beneficial genera. But there were still some shortcomings in our study. Fibrosis and granulomas were the typical symptoms and lethal causes of schistosomiasis japonica. Our study explained the effectiveness of *B. amyloliquefaciens* intervention for alleviating schistosomiasis japonica based on the results of alterations in the gut microbiome, but mechanisms remained unexplained. In our future studies, we expect to explore the mechanisms of alleviating pathological responses. Multi-omics analysis techniques such as metagenomics and metabolomics will be used to analyze the effects of *B. amyloliquefaciens* intervention on the metabolic pathways and metabolites of the intestinal microbiome, observe the changes in expression of inflammatory factors through measurement, and elucidate how the intervention of *B. amyloliquefaciens* could alleviate pathological damage by modifying the intestinal microenvironment from the perspective of the gut–liver axis. The current treatment with probiotics has been widely used clinically to treat IBD, but it is rarely used in the treatment of schistosomiasis japonica. For regulating host immunity by means of killing pathogens and secreting metabolites, probiotics have great prospects for schistosomiasis control (Yadav et al., 2022).

In conclusion, our results indicated that treating and alleviating schistosomiasis japonica by using *B. amyloliquefaciens* may prove to be an effective method. The establishment of early colonization with probiotics can reduce the pathological symptoms in mice infected with *S. japonicum*. Meanwhile, it can reshape the intestinal microenvironment of infected mice by modulating the relative abundance of potentially beneficial and harmful bacteria as well as the network of interactive relationships of the intestinal microbiome. These results also indicated that there is a potential relationship between pathological damage and changes in the gut microenvironment. Our study provided potential auxiliary therapeutic strategies and means for the early prevention and treatment of schistosomiasis japonica.

## Data availability statement

The data presented in the study are deposited in the Sequence Read Archive (https://www.ncbi.nlm.nih.gov/sra), under accession number PRJNA937169 (https://www.ncbi.nlm.nih.gov/bioproject/PRJNA937169/).

## Ethics statement

The studies involving human participants were reviewed and approved by The IRB of School of Basic Medical Science of Central South University (No: 2021-KT75). The patients/participants provided their written informed consent to participate in this study.The animal study was reviewed and approved by The IRB of School of Basic Medical Science of Central South University (No: 2021-KT25).

## Author contributions

JH and SH conceived the study. JW isolated and characterized *B. amyloliquefaciens*. HC and SY performed the experiments. HC and RS analyzed the data. HC and ZY visualized the statistical results. HC wrote the draft. HC, SH, and JH wrote and edited the final manuscript. SB improved the language and grammar of the manuscript. All authors contributed to the article and approved the submitted version.
