# Supplementary material for: Bacillus amyloliquefaciens alleviates the pathological injuries in mice infected with Schistosoma japonicum by modulating intestinal microbiome
Source: Front Cell Infect Microbiol. 2023 May 17;13:1172298. doi: 10.3389/fcimb.2023.1172298 (PMC10230073; doi:10.3389/fcimb.2023.1172298)
Supplement: Supplementary file 1 [file DataSheet_1.docx]

Supplementary Material

*Bacillus amyloliquefaciens* alleviates the pathological injuries in mice infected with *Schistosoma japonicum* by modulating intestinal microbiome

**Hao Chen^1,2^, Ruizheng Sun^3^, Jingyan Wang^2^, Siqi Yao^2^, Zheng Yu^2^, Shuaiqin Huang^1*^, Jing Huang^1, 2*^**

*** Correspondence:**

Jing Huang, jing_huang@csu.edu.cn; Shuaiqin Huang, sqhuang@csu.edu.cn

## Supplementary Figures


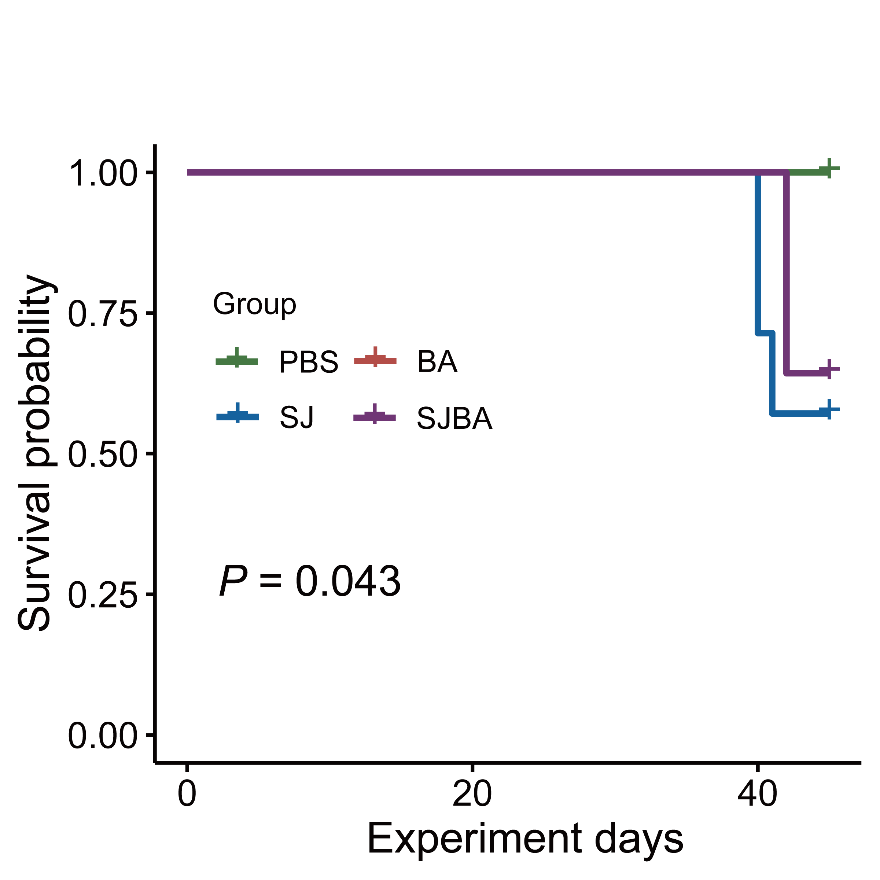


**Supplementary Figure S1 Survival curves of four groups of mice.**


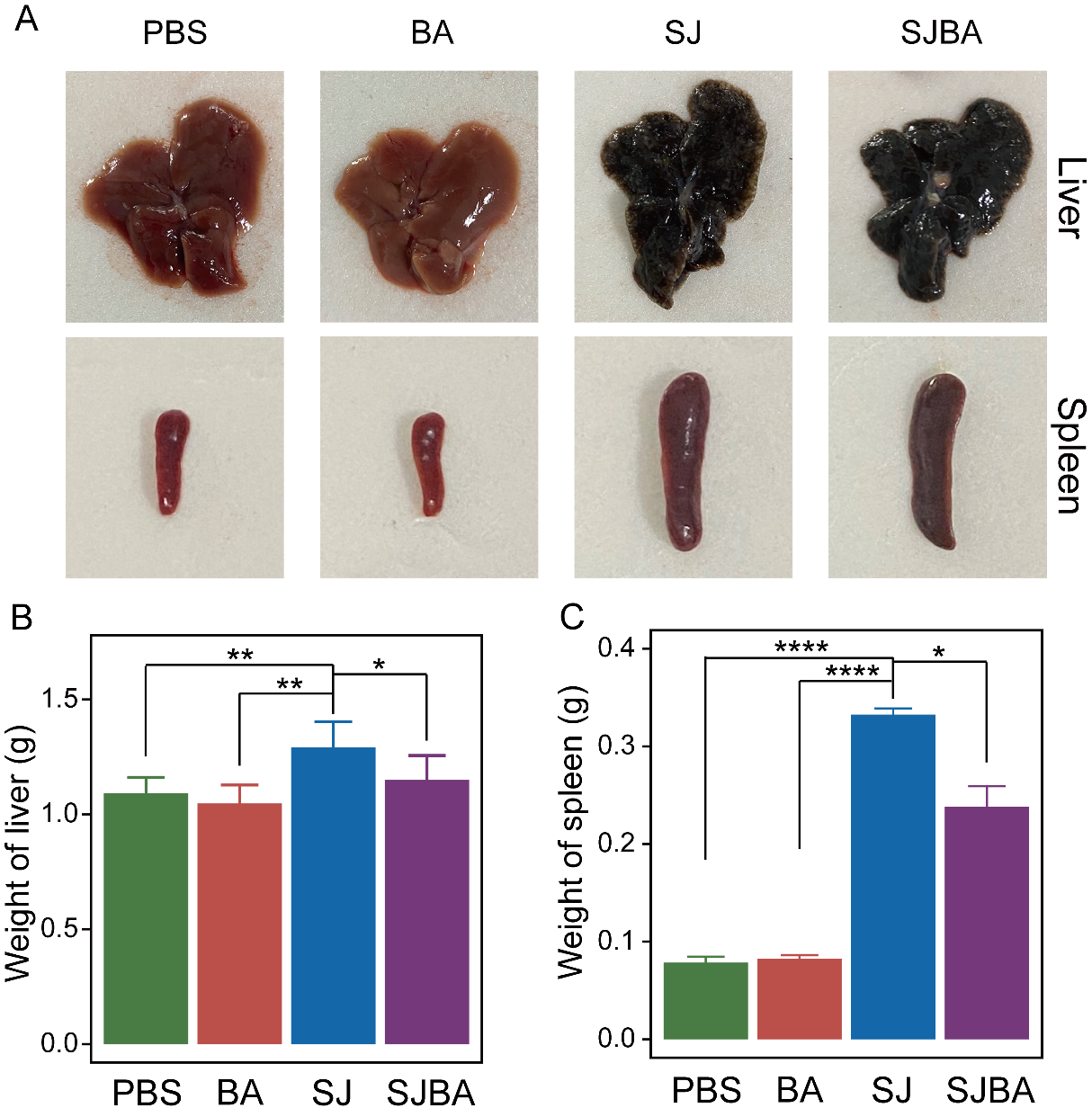


**Supplementary Figure S2 The changes of liver and spleen in *S. japonicum-*infected mice treated with *B. amyloliquefaciens*.** **(A)** The appearance changes of liver and spleen in mice. **(B)** The weight changes of liver in mice. Data were analyzed by one-way ANOVA and LSD’s multiple comparison posttest. **(C)** The weight changes of liver in mice. Data were analyzed by Kruskal-Wallis test. * *P* < 0.05; ** *P* < 0.01; *** *P* < 0.001; **** *P* < 0.0001


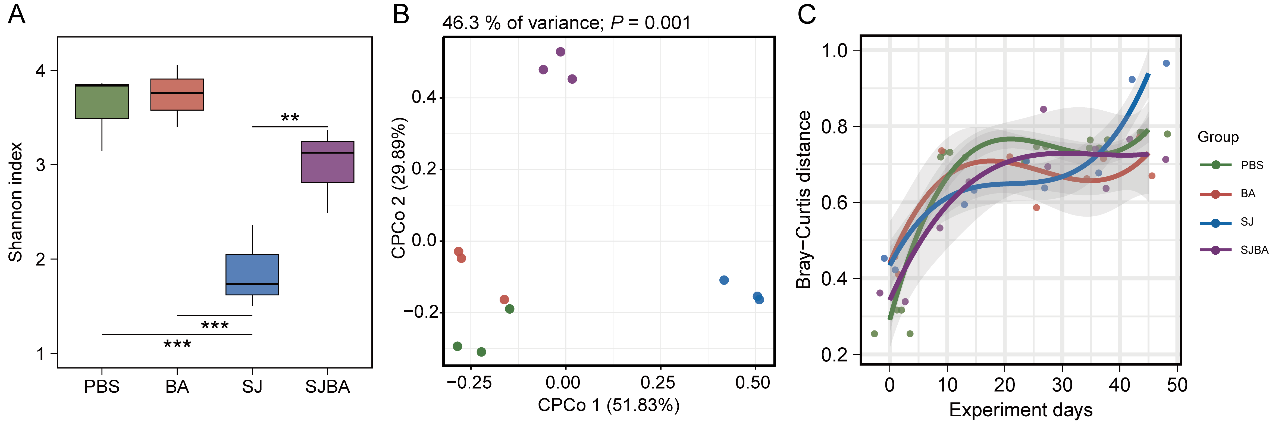


**Supplementary Figure S3 The changes of diversity in gut microbiome of *S. japonicum* infected mice treated with *B. amyloliquefaciens*. (A)** The changes of Shannon index of intestinal microbiome in mice of four groups on the 45^th^ day. Data were analyzed by one-way ANOVA and LSD’s multiple comparison posttest. **(B)** The analysis of CPCoA among intestinal microbiome in mice of four groups on the 45th day. **(C)** The changes of Bray-Curtis distances over the experimental days. * *P* < 0.05; ** *P* < 0.01; *** *P* < 0.001; **** *P* < 0.0001.


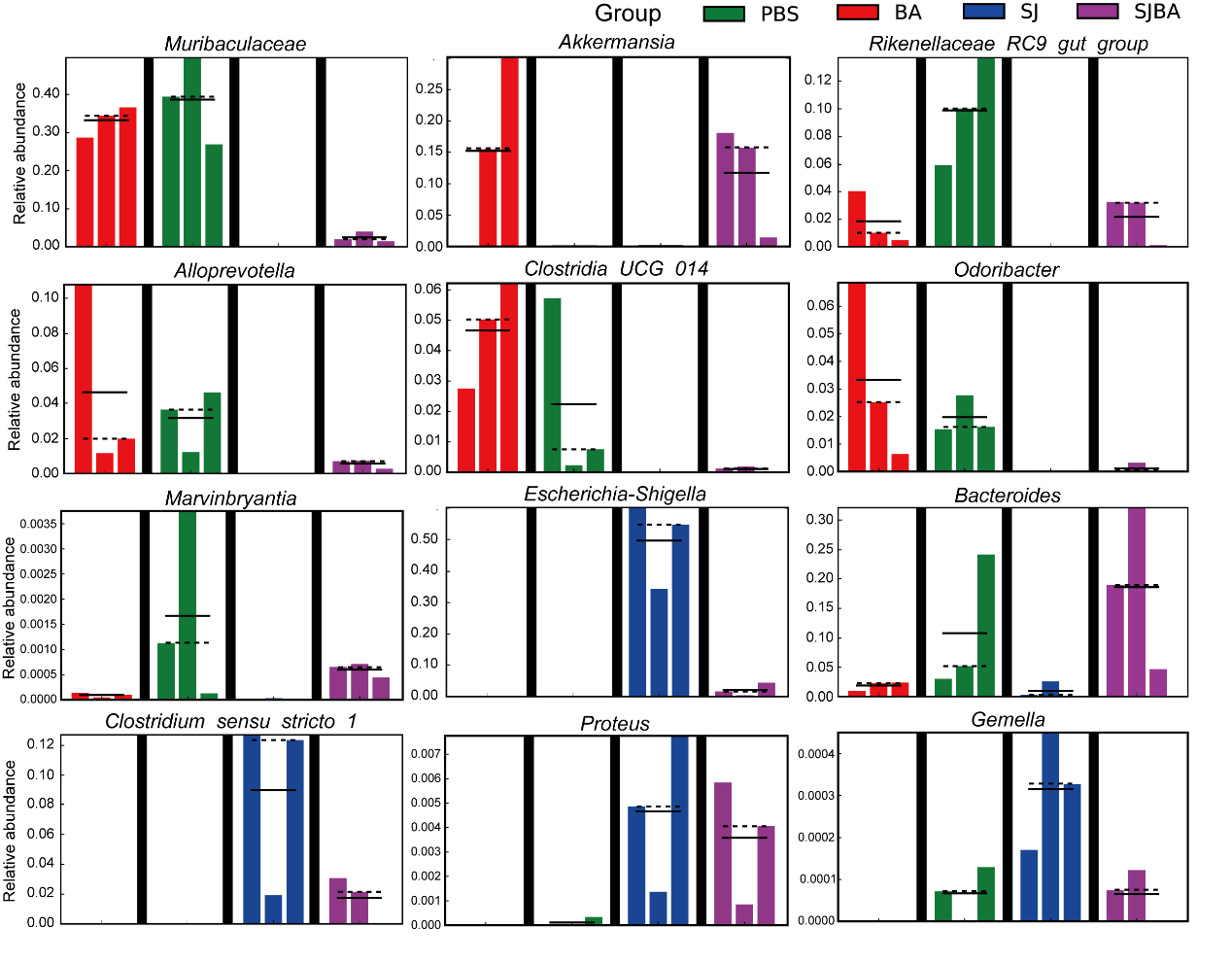


**Supplementary Figure S4 The changes of relative abundance in genera which has significant differences in four groups.**


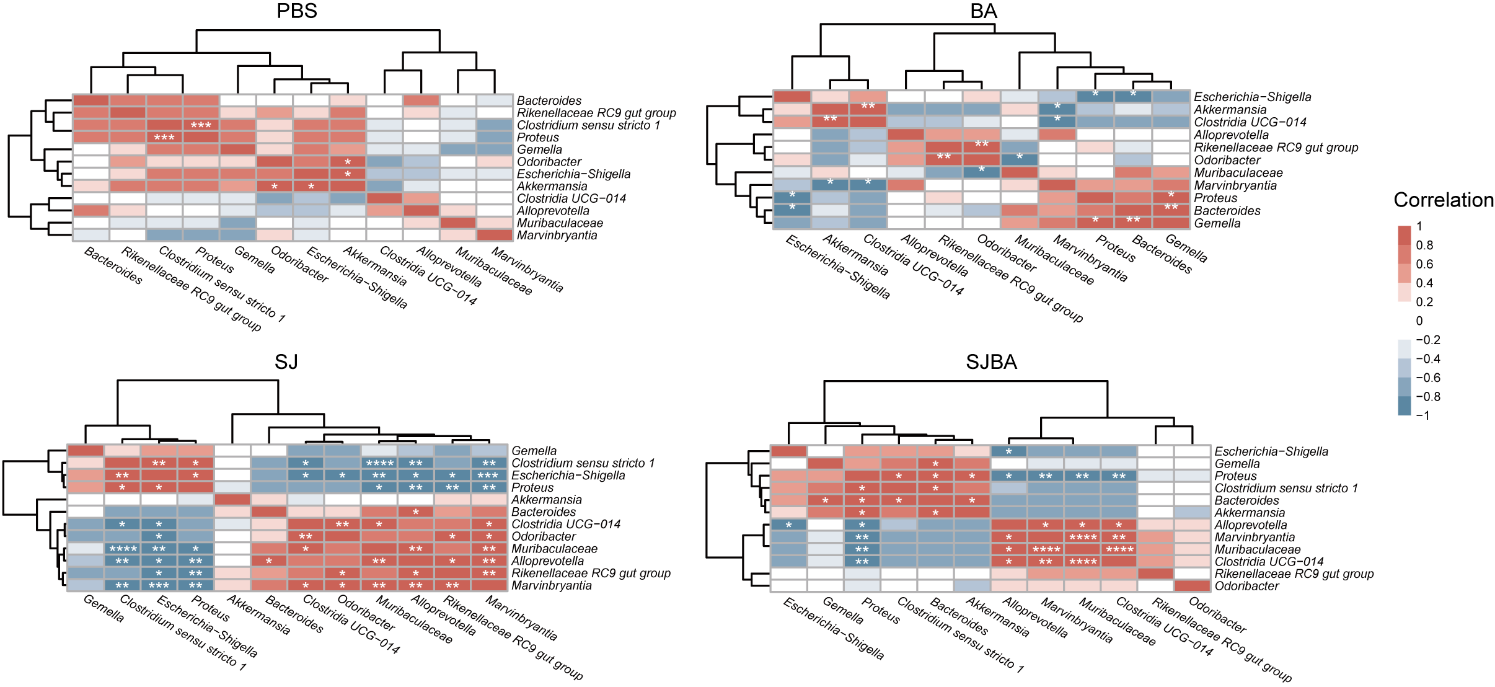


**Supplementary Figure S5 The analysis of Spearman correlation among all genera of significant difference in the relative abundance.** * *P* < 0.05; ** *P* < 0.01; *** *P* < 0.001; **** *P* < 0.0001.
